# Diffuse Gliomas with FGFR3-TACC3 Fusions: Oncogenic Mechanisms, Hallmarks, and Therapeutic Perspectives

**DOI:** 10.3390/cancers15235555

**Published:** 2023-11-23

**Authors:** Alberto Picca, Giulio Sansone, Orazio Santo Santonocito, Chiara Maria Mazzanti, Marc Sanson, Anna Luisa Di Stefano

**Affiliations:** 1 Paris Brain Institute (ICM), Sorbonne Université, Inserm, CNRS, UMR S 1127, 75013 Paris, Francemarc.sanson@aphp.fr (M.S.); 2 Department of Neuro-Oncology, Pitié-Salpêtrière University Hospital, Assistance Publique-Hôpitaux de Paris (AP-HP), 75013 Paris, France; 3 Neurology Unit, Department of Neuroscience, University of Padova, 35128 Padova, Italy; giuliosansonepd@gmail.com; 4 Division of Neurosurgery, Spedali Riuniti di Livorno, Azienda Sanitaria Toscana Nord-Ovest, 55100 Livorno, Italy; 5 Fondazione Pisana per la Scienza, San Giuliano Terme, 56017 Pisa, Italy; 6 Department of Neurology, Foch Hospital, 92150 Suresnes, France

**Keywords:** glioma, glioblastoma, gene fusions, *FGFR3*, *FGFR3*-*TACC3*, OXPHOS

## Abstract

**Simple Summary:**

Glioblastomas are the most common primary brain tumors in adults. They harbor a dismal prognosis and intrinsic resistance to treatment. Nonetheless, around 5% of glioblastomas present a unique genetic alteration, the FGFR3-TACC3 gene fusion, that drives tumor transformation and could represent a therapeutic opportunity. In this review, we discuss the state-of-the-art knowledge regarding glioblastomas with FGFR3-TACC3 gene fusions. We present their unique features and the methods for their identification. Targeted therapies aimed at inhibiting the protein resulting from the gene fusion have been developed with moderate clinical efficacy. An integrated effort is ongoing to improve the treatment results for these patients.

**Abstract:**

In 2012, whole-transcriptome sequencing analysis led to the discovery of recurrent fusions involving the *FGFR3* and *TACC3* genes as the main oncological driver in a subset of human glioblastomas. Since then, *FGFR3-TACC3* fusions have been identified in several other solid cancers. Further studies dissected the oncogenic mechanisms of the fusion protein and its complex interplay with cancer cell metabolism. *FGFR3-TACC3* fusion-driven gliomas emerged as a defined subgroup with specific clinical, histological, and molecular features. Several *FGFR* inhibitors were tested in *FGFR3-TACC3* fusion-positive gliomas and proved some efficacy, although inferior to the results seen in other *FGFR3-TACC3* fusion-driven cancers. In this review, we summarize and discuss the state-of-the-art knowledge resulting from a 10-year research effort in the field, its clinical implications for glioma patients, the potential reasons for targeted therapy failures, and the perspective of emerging treatments.

## 1. Introduction

*Isocitrate dehydrogenase* (*IDH*) wild-type (thereafter IDHwt) diffuse gliomas, of which glioblastoma (GBM) represents the most malignant and prototypical form, are the most frequent primary brain tumors in adults [1]. They are aggressive cancers with a limited response to current treatments and a consequently poor prognosis [2]. Nonetheless, IDHwt gliomas are a negatively defined (lack of IDH mutations), heterogeneous entity that could be further dissected. Indeed, about 3–5% of adult IDHwt diffuse gliomas bear an oncogenic chromosomal translocation involving the *fibroblast growth factor receptor 3* (*FGFR3*) and *transforming acidic coiled-coil protein 3* (*TACC3*) genes [3,4,5]. *FGFR3* is a component of the *FGFR* family of transmembrane receptor tyrosine kinases (RTK), which are physiologically involved in the regulation of several cellular processes, including development, survival, migration, and angiogenesis [6]. In physiological conditions, the binding of fibroblast growth factors leads to receptor dimerization and transphosphorylation of the intracellular TK domains. Downstream signaling results in the activation of the *RAS*/*mitogen-activated protein kinase* (*MAPK*), *phosphoinositide 3-kinase* (*PI3K*)/*Akt*, and *Signal transducer and activator of transcription 3* (*STAT3*) pathways [6]. An aberrant activation of FGFR signaling via missense mutations is seen in several cancers [7], including gliomas [8]. *TACC3* is a protein involved in mitotic spindle stabilization during cell division [9,10]. Both genes are closely located on chromosome 4p16 [11].

**Figure 1:**
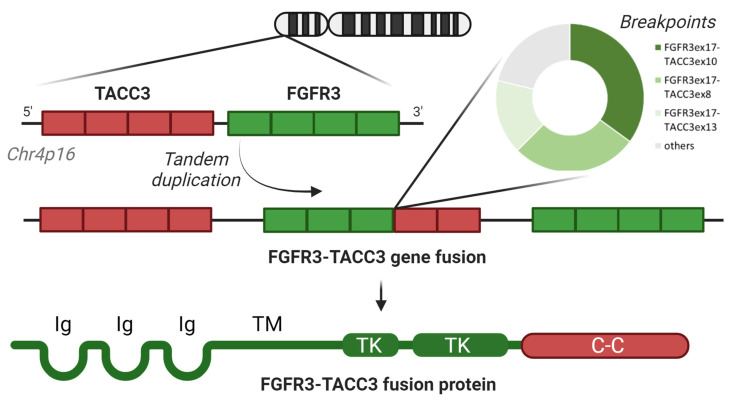
The structure of the genomic rearrangement and frequency of the different breakpoints in FGFR3-TACC3 fusions (upper part); the structure of the chimeric FGFR3-TACC3 fusion protein (bottom part). Ig = immunoglobulin-like domain; TM = transmembrane domain; TK = tyrosine kinase domain; C-C = coiled-coil domain. *Made with Biorender*.

In their first description by Singh et al. in 2012, *FGFR3-TACC3* (thereafter F3T3) fusions were detected from the transcriptome of cultured glioma cells obtained from nine GBM patients [3]. By detecting split reads and split inserts, the Authors discovered intrachromosomal rearrangements giving rise to in-frame fusion transcripts involving the N-terminus of FGFR3 and the C-terminus of *TACC3* [3]. In the predicted fusion protein, the intracellular tyrosine kinase (TK) domain of *FGFR3* was fused in-frame upstream to the coiled-coil (C-C) domain of *TACC3* (Figure 1). Subsequent works demonstrated that this fusion is the result of a tandem duplication of a 70 kb region on chromosome 4p16 (Figure 1), often accompanied by a low-level focal amplification [12]. Tumor cells presenting the gene fusion had an abundant expression of the chimeric protein [3].

Following these first reports, F3T3 fusions have been detected in several other cancers, including bladder [13,14], lung [14,15], cervical [16], nasopharyngeal [17], renal [18], and triple-negative breast [19] cancers. Although seen at a limited frequency (typically less than 10%) in specific tumor types, due to their widespread presence, F3T3 fusions could represent the most frequent gene fusion in solid cancers [11].

Additional *FGFR-TACC* gene fusions involving other components of the *FGFR* and *TACC* families that similarly present a close chromosomal location (*FGFR1* and *TACC1* on chromosome 8p11 and *FGFR2* and *TACC2* on chromosome 10q26 [11]) have also been discovered. *FGFR2*-*TACC2* fusions are recurrent in digestive cancers [20], and particularly in cholangiocarcinoma [21,22]. *FGFR1*-*TACC1* rearrangements are present in breast cancer [23] but also in low-grade gliomas [24,25]. *FGFR1*-*TACC1* gene fusions can be seen with a frequency of up to 60% in specific low-grade glioma entities such as extraventricular neurocytoma [26].

### 1.1. Screening and Identification of F3T3 in Gliomas

Because of the proximity of the *FGFR3* and *TACC3* genes on chromosome 4p16.3, the detection of F3T3 rearrangements by fluorescence in situ hybridization is not a feasible option. We previously developed an unbiased screening method [27], which includes a reverse transcriptase (RT)-PCR step with primers flanking *FGFR3* and *TACC3* regions that are retained in the fusion transcript; this allows the identification of known and novel F3T3 isoforms. RT-PCR amplification is followed by the confirmation of the presence of an in-frame breakpoint via Sanger sequencing [27]. A major drawback of this method is the need for frozen material. More recently, capture-based next-generation sequencing-based techniques have emerged, allowing for the robust detection of gene fusions also on paraffin-embedded specimens [28].

It was rapidly apparent that F3T3 GBMs display extensive positivity for immunohistochemical (IHC) staining using antibodies that recognize the N-terminal portion of *FGFR3*, always retained in the fusion protein [12,27]. Subsequent studies confirmed that the *FGFR3* IHC has a 100% sensitivity for F3T3 GBMs [4,29,30,31]. Nonetheless, molecular confirmation is required, as FGFR3 immunopositivity has a limited positive predictive value (about 25% in our experience [4]). Indeed, FGFR3 staining can also be positive in cases with FGFR3-nonTACC3 fusions [30,32] as well as other FGFR3 alterations such as gene amplification [32].

A cost-effective, time-saving approach for the prospective screening of F3T3 fusions in glioma patients includes a first step with *FGFR3* IHC. Its 100% negative predictive value [4] excludes negative cases from unnecessary molecular testing. If the IHC results are positive, the presence of an F3T3 fusion should be further confirmed by RT-PCR or NGS techniques, depending on the type of specimen (frozen or FFPE) and local availability. IHC prescreening of F3T3 fusions is part of the current EANO guidelines on molecular testing of gliomas [33].

### 1.2. Prevalence of F3T3 and Its Structural Variants in Gliomas

The first large screening for F3T3 fusions in glioma patients included 795 cases from different institutions [27]. In this study, our group found 20 patients with F3T3+ gliomas, of which seventeen were GBMs (prevalence of 2.9% in all GBMs), and three were histologically lower-grade IDHwt gliomas (3.5%) [27]. No F3T3 fusions were seen in *IDH* mutant glioma cases. In an expanded cohort of more than 950 unselected gliomas, we later confirmed a prevalence of 3–4% in both histologically grade 2–3 and grade 4 IDHwt diffuse gliomas [4]. A similar rate was seen in an independent study on 906 IDHwt GBMs, with 37 F3T3 positive cases (4.1%) [5]. No F3T3 fusion has been reported in *IDH* mutant gliomas so far.

The breakpoint position on the fusion protein appears to range widely [3,12,27]. Nonetheless, the fusion transcript invariably includes the intact *FGFR3* TK domain fused in-frame, with variable sequences of *TACC3* always retaining the C-C domain [27]. The fusion breakpoint is almost always located in exons 17 or 18 of the FGFR3 transcript, whereas greater variability is seen for TACC3 [4,5,31]. Three fusion isoforms (FGFR3ex17-TACC3ex11, FGFR3ex17-TACC3ex10, and FGFR3ex17-TACC3ex8) account for 80% of cases (Figure 1) [4], with in particular the first two prevailing across studies [5,31]. In rare cases, short intronic sequences are retained in the fusion transcript [4]; this seems required to maintain the reading frame when less common *TACC3* exons are involved in the fusion breakpoint. While a recent panglioma study (including circumscribed glioma entities such as ganglioglioma) suggests a better outcome for patients harboring the FGFR3ex17-TACC3ex10 isoform compared to FGFR3ex17-TACC3ex11 cases [31], this was not seen when the analysis was restricted to a more homogeneous cohort of IDHwt diffuse gliomas [4].

### 1.3. The Conundrum of Oncogenic Mechanisms of F3T3

We have clear evidence that the F3T3 gene fusion is highly oncogenic. F3T3-transfected cells proliferate and display anchorage-independent growth [3,12,13,34]. Transfected astrocytes form glioma-like tumors in immunodeficient mice [3]. Nonetheless, the exact oncogenic mechanisms of F3T3 are still not completely understood. The *FGFR3* TK and *TACC3* C-C domains are always retained in the fusion protein [27], suggesting that both are necessary for the generation of the oncogenic signal. Indeed, we have in vitro evidence that *FGFR3* TK activity is required for oncogenicity [3,34]. The presence of the C-terminal *TACC3* C-C domain induces protein dimerization with consequent transphosphorylation of key *FGFR3* tyrosine residues and constitutive activation of the FGFR TK [3,34,35].

The activation of canonical downstream signaling pathways of *FGFR3* (*MAPK*/*ERK*, *PI3K*-*Akt*, and *STAT*) remains controversial. In the first paper reporting the presence of F3T3, the authors did not detect increased phosphorylation of *ERK* and *Akt* proteins [3], suggesting a different mechanism behind the transforming properties of F3T3. Nonetheless, other groups illustrated that F3T3 leads to the activation of the *MAPK*/*ERK* and *STAT3* pathways but not *Akt* [12,34,36,37]. Immunohistochemistry showed phosphoERK and phosphoSTAT3 activity in F3T3+ tumors developing in the mouse brain [12].

Another matter of debate is the contribution of *TACC3* to the oncogenicity of F3T3. Indeed, even if *TACC3* is by far the most common (>80% of cases) gene partner in *FGFR3* rearrangements in cancer [38,39], several other partner genes have been detected [13,30,38,39]. Almost all (>95%) of the proteins encoded by these partner genes contain self-interacting domains, mostly C-C domains [39]. It is thus unclear whether *TACC3* serves merely as a donor for the self-interacting domain required for the constitutive activation of *FGFR3*, and its higher frequency could be explained by the proximity of the two genes on the genome and the presence of microhomology [3] (short DNA regions of identical sequence, a well-defined feature associated with the occurrence of gene fusions [40]), or if TACC3 actively takes part in the oncogenic signaling.

In this regard, Singh et al. noticed that the *TACC3* component could drive the intracellular location of the fusion protein, with F3T3 positioning at spindle poles at metaphase and relocating to the midbody in the late stages of mitosis [3]. This could lead to chromosomal instability and aneuploidy, contributing to the oncogenic transformation [3]. Subsequent experiments on HeLa cells did not detect mitotic spindle compartmentalization of the fusion protein but suggested that the F3T3 protein could reduce the levels of wild-type *TACC3* protein at the mitotic spindle via the binding to the C-C domain present in the fusion protein and subsequent sequestration [41]. The impaired function of wild-type *TACC3* would eventually result in chromosome segregation errors and aneuploidy [41]. It is relevant to note that F3T3 GBMs do not display an increased burden of copy number alterations compared to other GBMs [5].

Subsequent works showed that the F3T3 protein can relocate to the nucleus, with the presence of the C-C domain of *TACC3* being responsible for its nuclear localization [34]. This delocalized, constitutionally activated kinase could interact with novel substrates that lead to cancer progression [11,34]. This hypothesis is counteracted by the evidence that a Nuclear Localization Signal (NLS)-F3T3 construct with constitutive nuclear localization lacks oncogenic potential [36]. Conversely, in interphase, the fusion protein presents in vesicle-like formations consistent with a transmembrane protein [36]. The addition of an N-terminal myristylation sequence that directs the fusion protein to the plasma membrane restores *MAPK* pathway activation and oncogenicity, suggesting that membrane localization and canonical *FGFR3* downstream pathways are required for the oncogenic function of F3T3 [36].

Another putative mechanism contributing to the oncogenicity of the fusion protein is the loss of the terminal part of the *FGFR3* transcript [12,39,42]. The presence of a C-terminally truncated protein, lacking exon 18, has been linked to the emergence of the oncogenicity potential of *FGFR2*, regardless of the presence of a gene fusion partner with self-dimerization properties [42]. More recent results presented in abstract form suggest that, unlike *FGFR2*, both the loss of exon 18 and a fusion partner with self-interacting properties are required for *FGFR3* oncogenicity [39]. The exact domains responsible for the emergence of oncogenicity after the loss of FGFR exon 18 are not yet characterized. Truncation of the C-terminus also causes the loss of the regulatory 3′ Untranslated Transcribed Region (3′-UTR) of *FGFR3* in the fusion transcript. In normal conditions, *FGFR3* expression is negatively regulated by microRNA mirR-99a, which is highly expressed in the brain. F3T3 escapes miR-99a regulation via 3′-UTR loss [12]. However, the loss of FGFR3ex19 and the following 3′-UTR is only weakly oncogenic in vitro [13], arguing against a major role in the transforming potential of the F3T3 fusion protein.

A crucial step in the downstream signaling of F3T3 appears to be the phosphorylation of *Peptidylprolyl Cis/Trans Isomerase*, *NIMA-Interacting 4* (*PIN4*) [35]. Phospho-PIN4 fuels the protein synthesis necessary for tumor growth. Phosphorylated *PIN4* results in peroxisome biogenesis and the accumulation of reactive oxygen species (ROS) [35]. ROS accumulation is sensed by the *Peroxisome proliferator-activated receptor gamma coactivator 1-alpha* (*PGC-1α*)-*Estrogen-related receptor γ* (*ERRγ*) complex, which acts as a master regulator of mitochondrial biogenesis. This rewires the metabolism of the cancer cells, eventually inducing the activation of mitochondrial metabolism and oxidative phosphorylation (OXPHOS) in F3T3 cancers [35].

## 2. The Hallmarks of F3T3 Glioblastomas

**Figure 2:**
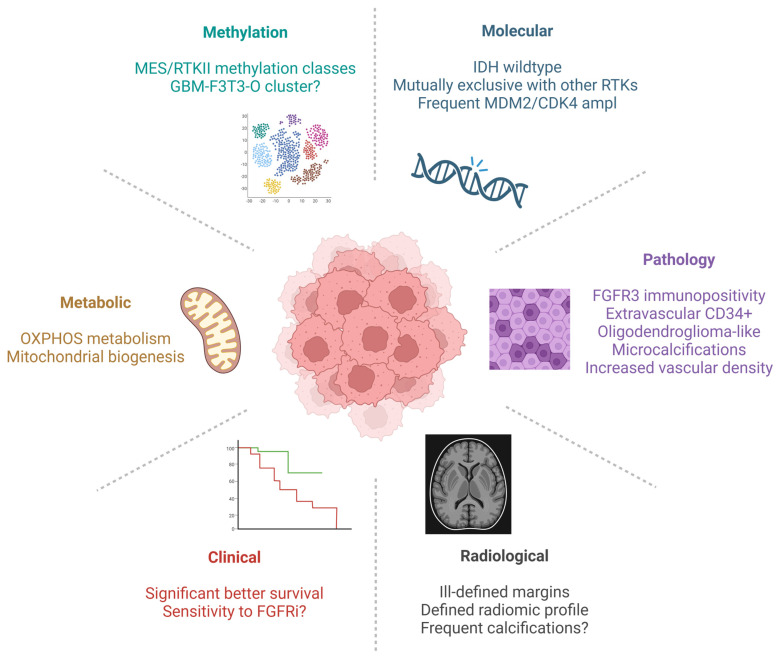
The “hallmarks” of FGFR3-TACC3 fusion-positive glioblastomas. *Made with Biorender*.

F3T3 GBMs display recurrent clinical, morphologic, and molecular features (Figure 2) that can help in their identification and inform patient prognosis.

### 2.1. Pathology

F3T3 GBMs display Olig2 and GFAP immunopositivity, maintained ATRX expression, and low p53 positivity. Extravascular CD34 positivity is present in more than half of cases [29,31], a feature that is shared with another tumor entity with recurrent F3T3 and FGFR2 fusions, the polymorphous low-grade neuroepithelial tumor of the young (PLNTY) [43]. As already mentioned, IHC targeting the N-terminus of FGFR3 is a useful tool for the identification and characterization of F3T3 gliomas [12,29,32]. F3T3 GBMs present a strong and diffuse FGFR3 immunopositivity [29,30] in accordance with the supposed clonal nature of the alteration. FGFR3 immunostaining allows the highlight of tumor cells, as the normal brain does not stain for FGFR3 (with the exception of the cerebellar and cerebral molecular layers, which weakly express FGFR3) [30]. Tumor cells display mainly cytosolic positivity, with rare nuclear FGFR3 staining [30,32].

Bielle and colleagues first reported that F3T3 GBMs present recurrent morphological features (RMF) [29]. These include monomorphic ovoid nuclei (oligodendroglioma-like), an endocrinoid (“chicken-wire”) network of thin capillaries, nuclear palisading, and thin parallel cytoplasmic processes forming vague pseudorosettes [29]. These characteristics are present at least focally in >70% of F3T3 GBMs [29]. Other recurrent features include microcalcifications and desmoplastic changes (that is, a proliferation of fibrous tissue with collagen deposition creating a fibrous, scar-like aspect) in more than 50% of cases [29,31]. RMF is more evident in tumor portions with lower-grade histology and can be lost in more malignant, necrotic compartments [29,44]. Subsequent works confirmed that RMF is frequently found in F3T3 GBMs [45,46], particularly in cases with lower-grade presentation [47]. Nonetheless, some F3T3 lack RMF [29,46,47]. RMF can also be found in GBM cases without F3T3 fusions [46] and other gliomas, in particular, some pediatric-type diffuse LGGs such as PLNTY and other MAPK-activated pediatric LGGs [31]. Recently, a “tissue culture-like” appearance composed of spindled neoplastic cells embedded in a loose, myxoid background has also been reported in a case of F3T3 GBM [48]. F3T3 GBMs have a significantly increased vascular density compared to fusion-negative GBMs [29,32]; this may be an expression of the increased oxygen consumption due to the activated mitochondrial metabolism [35].

### 2.2. Clinical Features

F3T3 GBMs show a male predominance and peak in the sixth and seventh decades of life [4,5], similarly to other GBMs. F3T3 GBM patients have better overall survival compared to fusion negative cases, consistently reported across studies [4,5,46]; the median overall survival ranged from 26.7 to 31.1 months in these series [4,5,46]. Molecular [4], metabolic [35,49], and methylation [50] features can further stratify F3T3 patients, as discussed in the following sections.

### 2.3. Radiological Features

We previously extensively explored the radiological profile of F3T3 GBMs [4]. F3T3 GBMs tend to have ill-defined tumor margins and reduced contrast enhancement [4]. They are preferentially located in the insula and temporal lobes [4]. On contrast-enhanced MRI, a specific radiomic profile may help to predict F3T3-positive cases [4]. Recently, it has been suggested that F3T3 gliomas have an increased incidence of macrocalcifications on CT scans [51], mirroring the frequent occurrence of calcifications seen on histopathological specimens [29].

### 2.4. Genetic Landscape

The molecular landscape of F3T3 GBMs is characterized by the absence of other truncal alterations, such as *IDH1/2* and *histone H3K27M* mutations [4]. F3T3 fusions are mutually exclusive with activating alterations of other RTKs often encountered in GBMs, such as *EGFR*, *PDGFRA*, *KIT*, and *MET* [4,12,30,31]. F3T3 GBMs have an increased incidence of focal amplifications on *MDM2* and/or *CDK4*, two oncogenes located closely on chromosome 12q13.15 [4,32]. Patients bearing *MDM2* and/or *CDK4* amplifications may have a better survival compared to other F3T3 GBMs [4]. Two cases presenting the co-occurrence of F3T3 and an *FGFR3* K650T activating mutation have been reported [45]; the real incidence among F3T3 gliomas of concurrent activating FGFR3 mutations and their prognostic value remains to be defined. Other recurrent alterations seen in GBMs, such as *pTERT* mutations, *CDKN2A* and *PTEN* inactivations, chromosome 7 gain, and chromosome 10 loss, are seen with frequencies comparable to F3T3 negative cases [5,27], with the exception of p53 mutations, which appear less frequent [5]. The hypermethylation of *MGMT* promoter, a marker of response to alkylating agents, is present in around 32–53% of cases [4,5]. As already mentioned, F3T3 GBMs display a slightly decreased rate of copy number alterations compared to other GBMs [5]. We recently detected the presence of chromosome 19 gain (alone or in association with chromosome 20 gain) as a marker of better survival among F3T3 GBMs [52].

### 2.5. Methylation Profiling

Recently, methylation profiling has emerged as a powerful tool for the identification of novel tumor entities in neuro-oncology [53,54]. Tumor methylation profiles are presumed to reflect both the cell of origin and the modifications induced by the oncological transformation. A methylation-based classifier of central nervous system tumors (the DKFZ classifier) has been developed [54] and recognized by the current WHO classification of brain tumors [1]. F3T3 GBMs are almost invariably assigned to mesenchymal or RTK II glioblastoma subclasses of the DKFZ classifier version 11 [5,31,50,52]. Recently, Wu et al. identified a small methylation cluster of molecular GBMs (i.e., with chromosome 7 gain, chromosome 10 loss, pTERT mutation) with F3T3 fusions but no necrosis, oligodendroglioma-like morphology, calcifications, a delicate vascular network, and a better outcome compared to other F3T3 GBMs (median overall survival around 40 months) [50]. These “outlier” F3T3 GBMs, or GBM-F3T3-O, had higher global DNA methylation compared to other GBMs and tended to be reassigned to the ganglioglioma methylation class by the 12.5 version of the DKFZ [50].

### 2.6. Metabolism

A recent pathway-based analysis of a single glioma cell transcriptome identified four cellular states distributed along metabolic and neurodevelopmental axes [49]. The metabolic axis, in particular, defines a mitochondrial (MTC) transcriptional state characterized by the activation of mitochondrial metabolism and OXPHOS functions in opposition to a glycolytic/plurimetabolic state enriched in multiple metabolic activities [49]. Transcriptome-based classification of bulk tumors could inform patient prognosis and potential therapeutic vulnerabilities. GBMs classified as MTC (upregulated mitochondrial metabolism) presented a significantly better outcome compared to the other classes [49], similar to what is reported for other systemic cancers [55]. A potential explanation may be the increased sensitivity of MTC cells to radiation therapy [49], a backbone of GBM treatment. As previously discussed, F3T3 GBMs display an energetic metabolism mainly based on cellular respiration and OXPHOS [35]. They overexpress mitochondrial markers such as *Voltage-dependent anion-selective channel 1* (*VDAC1*) and *NADH:ubiquinone oxidoreductase subunit S4* (*NDUFS4*) [35]. Indeed, F3T3 GBMs tend to be associated with the MTC class [56]. This association could at least partially explain the better survival of F3T3 GBM patients and represents a potential therapeutic target, as discussed below.

### 2.7. F3T3 as a Theranostic Marker?

The presence of F3T3 fusions as the main oncogenic driver in a subset of GBMs suggests a specific vulnerability of these tumors to FGFR inhibition. Indeed, the treatment with *FGFR* inhibitors (FGFRi) fexagratinib (AZD4547) or erdafitinib (JNJ-42756493) inhibits the growth of glioma stem cells expressing F3T3 at low nanomolar concentrations (<10 nM) [3,27]. Mice harboring F3T3 glioma xenografts have an increased survival when treated with FGFRi [3,27].

A crucial question for the effective clinical translation of any novel candidate target alteration is its frequency in the cancer cell population, thus discriminating between clonal and subclonal events [57]. In fact, GBM is typically characterized by a formidable degree of subclonal heterogeneity [58], with neighboring cells displaying activation of different RTKs [59]. In this regard, F3T3 GBMs appear of interest given the strong, homogeneous intratumor expression of the F3T3 fusion protein [29,30] and the mutual exclusion with other RTK aberrations [4,12,30,31]. Another cause of failure of molecularly driven treatments in GBMs is the temporal instability of the target, which can be lost at recurrence [60]. Analyses of paired primary–recurrent tumors found that F3T3 is retained at recurrence [27,30,44,61].

Given its actionability, F3T3 is acknowledged as a target of potential interest in current EANO guidelines [33]. On the ESMO Scale for Clinical Actionability of Molecular Targets (ESCAT) scale [62], it has been considered as IIB tier (availability of drugs with antitumor activity, but magnitude of benefit unknown) [33].

## 3. FGFR Inhibition in F3T3 Glioblastomas

The first evidence of an antitumor effect of FGFRi in F3T3 gliomas came from two patients with recurrent F3T3 GBM who were treated with erdafitinib (previously JNJ-42756493) in a first-in-human phase I trial [63]. Both patients manifested clinical improvement with disease stabilization (concomitant reduction in rCBV) in one and minor response in the other. Disease control lasted 115 and 135 days, respectively [27]. Several clinical trials have since been performed with anti-FGFR therapies, including patients with brain tumors harboring *FGFR3*-*TACC3* fusions (Table 1).

### 3.1. Erdafitinib

The first-in-human study (NCT01703481) with erdafinitib for patients with advanced solid tumors showed a stronger signal of activity in patients with FGFR1-4 gene fusions compared to patients with other alterations (gene amplifications or point mutations) [63]. Among the three patients with recurrent GBMs, one showed a RANO partial response [27,63]. The efficacy of erdafitinib on urothelial carcinoma seen in this trial was confirmed in a subsequent phase II study [70] that led to the FDA approval for this indication.

The subsequent phase II RAGNAR trial (NCT04083976) recruited patients affected by recurrent solid tumors (except for urothelial cancer) with FGFR1–4 mutations or fusions [64]. Data from RAGNAR confirmed the antitumor activity of erdafitinib across histologies, with an overall objective response rate of 30% [64]. Of the 217 patients treated, 30 were affected by high-grade gliomas presenting mostly FGFR3 gene fusions. Objective responses were seen in 10% of them: two patients with FGFR3-TACC3 fusions and one with an FGFR1-TACC1 fusion, including two cases still responding at the time of data cutoff more than 23 months from treatment initiation [64].

An NCI-sponsored phase II trial is planned to test erdafitinib specifically in patients with recurrent or progressive gliomas with FGFR-TACC fusions (NCT05859334)

### 3.2. Infigratinib

Infigratinib (BGJ398) is an oral inhibitor of FGFR1-3 tyrosine kinases. It has received FDA accelerated approval for the treatment of FGFR2-rearranged cholangiocarcinoma with FGFR2 rearrangements [71]. Infigratinib has been tested in patients with recurrent gliomas harboring FGFR1-4 alterations in a phase II trial (NCT01975701) [65]. The drug demonstrated a limited efficacy (objective response rate of 5%, 6-month progression-free survival rate of 16%). Efficacy results could have been underestimated given the initial inclusion of cases with isolated FGFR amplifications, whose oncogenic effect is not demonstrated. Durable disease control was seen in a subset of patients, including a patient with an F3T3 positive glioma that was still under treatment free from progression 30 months after inclusion [65].

### 3.3. Fexagratinib

Fexagratinib (AZD4547) is a potent oral FGFR1-3 inhibitor that demonstrated antitumor activity in preclinical models of F3T3 gliomas [3,27]. It has been tested in a phase I/II trial dedicated to recurrent gliomas harboring FGFR gene fusions (NCT02824133). Twelve patients with F3T3 gliomas (ten GBMs) were included. Results have been presented in abstract form at SNO 2023 [72]. The 6-month progression-free survival (PFS) was 25%, with a median PFS of 1.4 months. One delayed response was seen in a patient after 13 months of treatment. A trend toward better results for patients treated at first recurrence was also reported [72].

### 3.4. Pemigatinib

Pemigatinib (INCB054828) is an oral FGFR1–3 inhibitor. In the FIGHT-101 phase I/II study (NCT02393248), including refractory advanced malignancies, responses were seen in 12 (9%) of 128 patients, of whom five had cholangiocarcinoma. The following results confirming its activity in patients with cholangiocarcinoma [73] led to pemigatinib approval for this indication. No data are available on brain tumor patients in FIGHT-101, except for a reported partial response in a recurrent pilocytic astrocytoma harboring an FGFR1 N546K mutation [74]. In the subsequent phase II FIGHT-207 trial, thirteen patients with FGFR-altered gliomas were included, mostly affected by GBMs (*n* = 9), and F3T3 was the most common molecular alteration (*n* = 9). The results are available in abstract form [66,75] and display a promising antitumor activity of pemigatinib, with two disease stabilizations and two RANO responses (including a complete response) in the nine F3T3 patients. Full results are awaited. The currently recruiting phase II trial FIGHT-209 (NCT05267106) is dedicated to recurrent gliomas with FGFR1-3 alterations [76].

### 3.5. Zoligratinib

Zoligratinib (Debio-1347) is another oral ATP-competitive FGFR1-3 inhibitor; it has been tested in patients with FGFR1-3 fusion-positive advanced solid tumors in a phase I trial (NCT01948297). Among the 18 patients included in the expansion cohort, five presented with an F3T3-positive brain tumor. Disease control was obtained in 79% of patients with systemic tumors versus 0% in the brain tumor patients [67]. Primary brain tumors were therefore excluded from the subsequent phase II FUZE trial [77]. Five pediatric patients affected by FGFR-altered gliomas received zoligratinib outside a clinical trial. Among them, two had low-grade gliomas with FGFR-TACC fusions. A durable clinical benefit was seen in both cases, consisting of a partial tumor response lasting nine months in one case and a tumor stabilization persisting for more than two years in the latter [68]. There are no currently ongoing trials employing zoligratinib.

### 3.6. Futibatinib

Futibatinib (TAS120) is an irreversible FGFR1-4 inhibitor predicted to be more resistant to the emergence of resistance mutations compared to other ATP-competitive inhibitors. Futibatinib was tested in a phase I basket trial (NCT02052778) that included a brain tumor cohort [69]. Among the 170 patients with FGF/FGFR aberrations that received the currently recommended dose of 20 mg daily, 36 (21%) had CNS tumors, including 25 with FGFR-TACC fusions (23 FGFR3-TACC3, 2 FGFR1-TACC1). One patient with an FGFR1-TACC1 positive GBM experienced a partial response lasting 5.8 months [69]. Tumor shrinkage was seen in eight patients with F3T3+ CNS tumors. Better results were observed in patients affected by cholangiocarcinoma patients with FGFR2 alterations [78], for which the drug received accelerated approval.

In conclusion, FGFRi displays moderate antitumor activity in patients with F3T3 gliomas, particularly when compared to the remarkable results obtained in other FGFR-rearranged cancers such as urothelial carcinoma [70] or cholangiocarcinoma [71,73,78]. The reasons for this marked difference are not yet understood. A pharmacodynamic issue with limited access of the drug to target cells due to the blood–brain barrier is a possible explanation; a recent phase 0 trial assessed infigratinib concentrations in non-enhancing tumor regions of patients with recurrent FGFR-altered gliomas undergoing resurgery after a one-week treatment with the FGFRi [79]. The first results show suboptimal drug levels in the evaluated tissue [79]. Nonetheless, the sporadic but consistent responses seen across different FGFRi [27,64,65,69,75] demonstrate that these compounds can reach the target in at least a subset of cases.

## 4. New Approaches and Perspectives in the Treatment of F3T3 Glioblastomas

Given the limited results seen in clinical trials of FGFR inhibition in F3T3 gliomas, new approaches are actively investigated to improve the management of these patients.

The OXPHOS metabolism of F3T3 gliomas has emerged as a promising therapeutical target. The reliance of MTC tumors upon the OXPHOS metabolism as a source of energy [49] can represent a potential state of synthetic lethality. Indeed, different mitochondrial inhibitors reduce the viability of MTC cells in vitro [49]. The same effect is also seen specifically on F3T3 glioma cells [35]. Based on these preclinical results, a phase II trial is scheduled to test the efficacy of the well-tolerated, widely available mitochondrial inhibitor metformin in addition to standard first-line chemoradiation in patients with newly diagnosed OXPHOS GBMs (OPTIMUM trial, NCT04945148). Novel mitochondrial inhibitors are under investigation; among them, IACS-010759 is a potent complex I inhibitor. A phase I trial of IACS-010759 in patients with solid cancers (including four patients with GBM) has been recently discontinued due to limited oncological efficacy and severe dose-limiting toxicities [80]. Nanoparticles to direct the OXPHOS inhibitor Gboxin [81] to GBM cells have recently been developed [82].

There is little emerging evidence regarding the use of MEK inhibitors (MEKi) against the FGFR-dependent MAPK pathway activation in FGFR-altered gliomas. Trametinib reduces MAPK phosphorylation and focus formation in F3T3-transfected HEK cells [36]. The use of trametinib has been reported in two patients with FGFR-rearranged pediatric-type gliomas (one dysembryoplastic neuroepithelial tumor with an FGFR1 internal tandem duplication and one F3T3 diffuse low-grade glioma), resulting in tumor stabilization in both cases, with only minimal toxicity [83].

Innovating approaches proposed for the treatment of patients with F3T3 include RNA interference [84] and CRISPR-Cas13a [85] techniques targeting F3T3 fusion breakpoints. Both technologies have been shown to reduce tumor growth in in vitro and mouse models of F3T3 tumors [84,85]. Emerging nanotechnologies, such as nanocarriers for targeted drug delivery [86,87], could be useful in both improving FGFRi delivery to the brain and limiting systemic toxicity.

## 5. Conclusions

Gliomas harboring F3T3 fusions emerged as a distinct niche among IDH wild-type gliomas. They present peculiar features from the molecular (oncogenic pathways, distinct metabolism) to the macroscopic (clinical, histological, and radiological phenotypes) levels. More importantly, F3T3 fusions can be targeted with specific FGFRi. Such inhibitors have shown, so far, a signal of activity but overall moderate results in dedicated phase I and II clinical trials dedicated to the recurrent stage of the disease. However, given the lack of effective therapies for recurrence in this population, systematic screening for F3T3 gene fusions in newly diagnosed IDHwt diffuse glioma patients is warranted, as recently recommended by EANO guidelines (ESCAT level 2). Further efforts are ongoing to optimize the effectiveness of specific TK inhibitors and/or develop alternative therapeutic approaches, such as metabolic manipulation.

## Figures and Tables

**Table 1:** Selected results of FGFR inhibitors studies in glioma patients. F1T1 = FGFR1-TACC1 fusion; F3T3 = FGFR3-TACC3 fusion; GBM = glioblastoma; HGG = high-grade glioma; CNS = central nervous system; PR = partial response; CR = complete response; SD = stable disease; ORR = overall response rate; PFS = progression-free survival; mo = months.

Study	Study Design	Target Population	Selected Results
**Erdafitinib (JNJ-42756493)**			
NCT01703481 [63]	Phase I	Advanced solid tumors	3 brain tumor patients included. 1 PR in F3T3 GBM.
NCT04083976 (RAGNAR) [64]	Phase II	Advanced solid tumors with FGFR1-4 fusions or mutations	10 HGGs included. 3 PR (3 F3T3, 1 F1T1).
NCT05859334	Phase II	Recurrent gliomas with FGFR-TACC fusions	Ongoing, not yet recruiting.
**Infigratinib (BGJ398)**			
NCT01975701 [65]	Phase II	Recurrent gliomas with FGFR1-3 amplifications, fusions, or activating mutations	26 patients included, of which 10 with F3T3. ORR 5%, 6-mo PFS 16%. One prolonged response (32+ mo) in an F3T3 GBM
**Fexagratinib (AZD4547)**			
TARGET	Phase I/II	Recurrent gliomas with FGFR gene fusions	12 F3T3 patients included. ORR 8% (1 delayed PR after 13 mo of treatment), 6-mo PFS 25%
**Pemigatinib (INCB054828)**			
NCT03822117 (FIGHT-207) [66]	Phase II	Advanced solid tumors with FGFR1-4 fusions or mutations	13 patients with FGFR-altered gliomas included (9 with F3T3). 1 CR, 1 PR, 2 SD in the 9 F3T3
FIGHT-209 NCT05267106	Phase II	Recurrent gliomas with FGFR1-3 alterations	Ongoing, recruiting
**Zoligratinib (Debio-1347)**			
NCT01948297 [67]	Phase II	Advanced solid tumors with FGFR1-3 fusions	Five brain tumor patients included, 0% disease control
[68]	Single-patient use protocols	Pediatric FGFR-altered gliomas	Five patients, including 1 F3T3 and 1 F1T1. In FGFR-TACC fusions, 1 PR and 1 sustained SD
**Futibatinib (TAS120)**			
NCT02052778 [69]	Phase I	Advanced solid tumors	36 CNS tumors (23 F3T3 fus, 2 F1T1 fus). 1 PR in F1T1, 8 tumor volume reductions in F3T3
